# Soda and Tobacco Industry Corporate Social Responsibility Campaigns: How Do They Compare?

**DOI:** 10.1371/journal.pmed.1001241

**Published:** 2012-06-19

**Authors:** Lori Dorfman, Andrew Cheyne, Lissy C. Friedman, Asiya Wadud, Mark Gottlieb

**Affiliations:** 1 Berkeley Media Studies Group, Berkeley, California, United States of America; 2 Public Health Advocacy Institute, Boston, Massachusetts, United States of America

## Abstract

In an article that forms part of the *PLoS Medicine* series on Big Food, Andrew Cheyne and colleagues compare soda companies' corporate social responsibility (CSR) campaigns - which are designed to bolster the image and popularity of their products and to prevent regulation - with the tobacco industry's CSR campaigning.

**Summary Points**

- Because sugary beverages are implicated in the global obesity crisis, major soda manufacturers have recently employed elaborate, expensive, multinational corporate social responsibility (CSR) campaigns.
- These campaigns echo the tobacco industry's use of CSR as a means to focus responsibility on consumers rather than on the corporation, bolster the companies' and their products' popularity, and to prevent regulation.
- In response to health concerns about their products, soda companies appear to have launched comprehensive CSR initiatives sooner than did tobacco companies.
- Unlike tobacco CSR campaigns, soda company CSR campaigns explicitly aim to increase sales, including among young people.
- As they did with tobacco, public health advocates need to counter industry CSR with strong denormalization campaigns to educate the public and policymakers about the effects of soda CSR campaigns and the social ills caused by sugary beverages.

*This article was commissioned for the* PLoS Medicine *series on Big Food that examines the activities and influence of the food and beverage industry in the health arena.*

For the first time in 23 years, PepsiCo eschewed the “biggest marketing day of the year” and did not advertise during the 2010 Super bowl [1],[2]. Instead, it launched the Pepsi Refresh Project, a social media cause marketing campaign. The campaign signaled a landmark turn in soda marketing, using cutting-edge social media techniques [3] to spread word-of-mouth buzz and elicit online nominations for a variety of community-based projects. In 2010, Pepsi donated more than $20 million to support causes that received the most votes, and intends to transform the Refresh Project into a global phenomenon [4]. Meanwhile, industry leader Coca-Cola maintains Live Positively, another corporate social responsibility (CSR) campaign that offers consumers healthy lifestyle advice and touts the firm's philanthropic and sustainability efforts.

Both companies' campaigns occur amidst increasing pressure from consumers and public health advocates concerned about rising obesity rates [5],[6], including the passage or consideration of strong legislative measures such as food taxes in many countries [7],[8],[9],[10]. While tobacco-related diseases remain a top public health threat [11], obesity is the fifth leading mortality risk worldwide [12], and the spread of western diets is expected to exacerbate preventable chronic conditions such as cardiovascular disease [13] and diabetes [14]. Globally, childhood obesity is “one of the most serious public health challenges of the 21st century” [15]. Sugar-sweetened beverage (SSB) consumption has helped fuel this crisis [16],[17]; from 1977 to 2004 U.S. children more than doubled their caloric intake from SSBs, in 2004 they received 13% of their caloric intake from SSBs [17], and these drinks have contributed an estimated one-fifth of the weight gain in the U.S. population from 1977 to 2007 [18].

When facing crises over health concerns, many industries attempt to thwart regulation and gain popular support [19]. The tobacco industry [20] has a long history of influencing the public and policymakers, and oil companies, among others, have emulated Big Tobacco's “playbook” in this regard [21],[22]. Wiist [23] explains how corporations aim to do this by distorting science, wielding political influence, deploying financial tactics, influencing legal and regulatory actions, promoting their own products and services, and investing heavily in public relations. Provocative comparisons of Big Tobacco and the food industry suggest that food companies may be using at least one of these tactics, specifically attempts to influence government policy, with similar aims [23],[24].

CSR is another of these corporate tactics. CSR has been defined as an evolving concept that has come to include companies' economic, legal, ethical, and philanthropic responsibilities to society in addition to the company's fiduciary responsibility to shareholders [25],[26]. Proponents of CSR argue it can help companies meet these essential needs while addressing the firm's “higher” social responsibilities [26]. Companies invest in CSR to address social demands; in an attempt to be accountable to groups beyond their shareholders, they accept ethical obligations to society at large [27]. Cause marketing is a variation of CSR that links the marketer to a specific social benefit, often a community initiative or organization that benefits from the sale of a product or brand [28].

Critics, however, portray CSR as primarily a public relations strategy designed to achieve “innocence by association” as corporations align themselves with good causes to burnish their public image and protect their core business [29],[30]. Corporations may use CSR to improve their standing among consumers, the press, legislators, and regulators who make policy decisions about the company and its products [27],[31],[32]. CSR initiatives are often introduced when corporations fear a threat to their profitability [33], because CSR can boost a firm's bottom line both directly through sales and indirectly by moderating the risk for regulation and improving the overall business climate.

After first reviewing an emblematic tobacco CSR campaign, we examine prominent cases from recent CSR efforts by soda industry leaders PepsiCo and Coca-Cola, to compare how these two industries have implemented CSR strategies.

## How Did Tobacco Companies Employ CSR?

During the 1950s, landmark scientific studies linked smoking and disease, and popular media disseminated the research [34]. The tobacco industry and its products began to suffer from reduced social acceptability and were targeted for tighter state and federal regulation [35],[36]. By the late 1990s, tobacco companies faced a series of challenges, including disclosures from industry whistleblowers and formerly secret internal documents, congressional hearings, a civil racketeering lawsuit by the U.S. Department of Justice, and the Master Settlement Agreement (MSA) with 46 state attorneys general compensating the states for Medicaid payments resulting from smoking-related illnesses.

Reacting to these pressures, the tobacco companies all began to implement CSR programs to improve their corporate and product images and to prevent legal and regulatory action [37],[38],[39]. In 1999, industry leader Philip Morris (PM) launched the industry's most ambitious and visible CSR program, which it internally labeled “PM21” [40]. In confidential documents, PM described the program as “a multi-faceted, cross functional effort to change the public's perception of Philip Morris and to improve the public's attitudes toward the company and the people who work for it” [41]. Using paid advertisements and a dedicated website, PM21 highlighted the company's charitable contributions to causes including homelessness, domestic abuse, and the arts [42],[43],[44]. This continued a previous strategy to co-opt interest groups that might oppose tobacco industry programs [45],[46],[47],[48],[49],[50]. While the PM21 campaign improved outlooks among the small segment of the public that had no pre-existing opinions about the company, the campaign hardened the opinions of the large majority who already held negative views of PM and the tobacco industry [42],[51].

PM21 was far from Big Tobacco's only CSR effort. The tobacco companies also launched CSR activities to protect areas of perceived vulnerability, which included regulation [52], litigation [20],[36], and future threats to their bottom line [53], such as declining social acceptability, youth smoking and concern over secondhand smoke exposure. In response to the prevalence of underage smoking, all of the major tobacco companies instituted “youth smoking prevention” programs to avert increased regulation [54],[55],[56]. For instance, PM distributed to students book covers emblazoned with the corporate name, and Lorillard employed the slogan “Tobacco Is Whacko If You're a Teen,” which emphasized the forbidden fruit aspect of youth smoking. Public officials, advocates, teachers, and students opposed these programs, which backfired because they were perceived as cynically employing reverse psychology to *encourage* youth smoking [57],[58],[59]. Through denormalization tactics that publicly exposed the tobacco industry's bad corporate behavior, tobacco control advocates joined with educators and elected officials to pressure the tobacco companies to drop their disingenuous “youth smoking prevention” programs.

## Snapshots of Soda Company CSR and Cause Marketing Campaigns

CSR and cause marketing have become industry-wide practices, including all leading SSB firms: Nestle [60], PepsiCo, Coca-Cola, and Dr. Pepper-Snapple Group [61]. Using information from the companies' campaign, corporate, and partner websites; their annual CSR reports; news and trade press coverage of the campaigns; and other reports, we examine prominent campaigns from industry leaders PepsiCo and Coca-Cola, which have embraced CSR with elaborate, expensive, and multinational campaigns [62],[63]. See a list of soda industry CSR-related URLs in Box 1.

**Box 1. Internet Presence of Soda Industry CSR Campaigns**

**PepsiCo CSR Campaigns**

- Refresh Project homepage: http://www.refresheverything.com
- Refresh Project Facebook: www.facebook.com/apps/application.php?id=301917354154
- Refresh Project Twitter: http://twitter.com/#/pepsi/pepsirefresh
- Shiv Singh, PepsiCo's Global Head of Digital, on the Refresh Project as marketing, including to youth: http://www.youtube.com/watch?v=Xb9Kby9_NBQ
- UK NHS on PepsiCo UK's Change4Life partnership: http://www.nhs.uk/change4life/Pages/national-partners-pepsico.aspx
- PepsiCo UK on Change4Life role: http://www.pepsico.co.uk/our-company/media-centre/news-and-comment/pepsico-uk-partners-with-department-of-healths-play4life-campaign

**Coca-Cola CSR Campaigns**

- Live Positively homepage: http://www.livepositively.com
- Sprite's Spark Your Park: http://www.livepositively.com/en_us/spritesparkparks/

**Other Sugary Drink Manufacturer's CSR Campaigns**

- Nestlé: http://www.nestle.com/CSV/Pages/Homepage.aspx
- Dr. Pepper Snapple Group: http://www.drpeppersnapplegroup.com/values/sustainability/

## PepsiCo's Refresh Project and Change4Life

The Pepsi Refresh Project dominates PepsiCo's CSR efforts in the U.S. The $20 million cause marketing campaign uses social media to identify philanthropic ventures [64]. Anyone may submit an idea online for a project, and PepsiCo funds the projects that generate the most votes each month, from community arts to “refreshing” parklands. Votes are cast on the campaign website, on its Facebook page, and on mobile devices via SMS messaging [65]. In January 2011, the Project explicitly linked the campaign to product sales by offering participants up to 100 additional “Power Votes” when they purchase specially marked PepsiCo beverages [66]. Globally, PepsiCo launched “Project Refresh,” which funds individual youth's ideas to make “the world more exciting and fun” in at least 18 countries from Venezuela to Ukraine [67].

The Refresh Project directly involves youth in PepsiCo's CSR campaign. PepsiCo has donated branded soda company items to a variety of youth-oriented causes such as children's ball fields [68] and band uniforms [69]. PepsiCo also hired a marketing firm to conduct a multi-city tour featuring popular musicians to inform youth about the initiative and to encourage them to submit grant proposals [70]. The Refresh Project successfully targeted Millennials—those currently aged 11–31 [71]—using traditional media, such as television, and new media, such as mobile devices, to drive “referral” marketing by leveraging Millennials' social networks [72].

Instead of separating moneymaking ventures from charitable donations, the contemporary soda industry CSR blurs the traditional lines between a corporation's profit-oriented and philanthropic activities. According to Shiv Singh, a marketing officer for the Refresh Project, the campaign is “not a traditional non-profit corporate philanthropy effort that we just go write checks. It's putting the DNA of doing and feeling good at the core of a brand marketing effort” [73]. Moreover, while the initiative is publicly presented as supporting charitable causes, the program was not funded with “corporate philanthropy dollars” but with “brand marketing dollars, because we believed fundamentally and still do that, you know, by doing good in a way that's aligned with our Pepsi brand values, you know, we can help the bottom line” [73]. By this, PepsiCo intends to take advantage of Millennials' desire to support or do business with companies that contribute to society [74] by associating their brand with all of the community projects they fund. PepsiCo considers Millennials a “key cohort” for the initiative, tracked their engagement with the campaign via new media, and used specific metrics to measure the positive effect the campaign had on their intent to buy PepsiCo products [75]. Accordingly, PepsiCo is using CSR as a marketing tool [73],[76],[77], in part to influence Millennials by reinforcing the view that it is a good corporate citizen.

Since 2009, PepsiCo has also been a partner with the United Kingdom National Health Service's Change4Life campaign. PepsiCo contributes to the “marketing component” [78] of that government's response to obesity, which promotes physical activity and healthy eating through traditional and new media social marketing campaigns. A “commercial partner” of the campaign, PepsiCo sponsored a major print ad buy for Change4Life that used famous soccer players to encourage parents to help their children “have an active lifestyle” [79].

## Coca-Cola's Live Positively

Coca-Cola's U.S. CSR activities occur under the Live Positively banner. They use educational campaigns such as “Balanced Living” or “Exercise is Medicine” to urge individual consumers to achieve healthy lifestyles; support charitable projects, such as the $2 million Spark Your Park (also called Sprite Spark Parks) initiative to refurbish basketball courts and school athletic fields in underserved communities [80]; and develop initiatives to improve the company's own business practices, e.g. reducing its water consumption. Coca-Cola promotes Live Positively through a dedicated website, full-page newspaper ads, more prominent nutrition labeling on product packaging, and a new line of 7.5-ounce “mini-cans.” Live Positively builds on Coca-Cola's existing CSR initiatives, such as the company's associations with youth organizations, including Coca-Cola's relationship with the Boys and Girls Club of America dating back 65 years [81].

Even from these brief descriptions it appears that the soda CSR campaigns reinforce the idea that obesity is caused by customers' “bad” behavior, diverting attention from soda's contribution to rising obesity rates. For example, CSR campaigns that include the construction and upgrading of parks for youth who are at risk for diet-related illnesses keep the focus on physical activity, rather than on unhealthful foods and drinks. Such tactics redirect the responsibility for health outcomes from corporations onto its consumers, and externalize the negative effects of increased obesity to the public [82],[32].

## Soda and Tobacco CSR: How Do They Compare?

Soda CSR campaigns echo tobacco CSR in their focus on the consumer and in their likely intent to thwart regulation. Soda CSR differs from tobacco in its explicit appeals to youth and in the aggressive launch of comprehensive campaigns soon after soda was linked to obesity.

## Soda and Tobacco CSR Shifts Responsibility from the Corporation to Consumers

By highlighting the importance of consumers making healthy choices instead of the companies' roles in creating an unhealthy environment, soda company and tobacco industry CSR campaigns emphasize personal, instead of corporate, responsibility. For instance, the tobacco industry's “youth smoking prevention” programs appeared to combat youth smoking, but instead placed responsibility on parents and children for the decision to smoke [55],[56]. Similarly, in its “Balanced Living” message on Live Positively, Coca-Cola suggests that the company is responsible only for providing health information to consumers, such as through the “Clear on Calories” labels that show calorie counts on the front of bottles or cans. The company suggests that health is ultimately up to consumers, because with new labels, “you'll know exactly how many calories are in a beverage before making a purchase—whether at a store, one of our vending machines or fountain machines—making it easier for you to make informed choices and live a healthy, active lifestyle” [83]. PepsiCo's advertisement for the UK's Change4Life campaign likewise insists that “active parents make active kids” [84].

## Tobacco and Soda Tactics Seek to Prevent Regulation

As CSR campaigns can improve a firm's standing with the public and policymakers, they are potentially a powerful mechanism to forestall regulation [47]. British American Tobacco, for example, used CSR to reestablish political influence with the UK Department of Health, with which its relationship had deteriorated [85]. While the Refresh Project and Live Positively have not stated such goals outright—and we have no cache of internal soda industry documents to investigate for such explicit rationales—the campaigns employ the very tactics that companies use to influence the public and policymakers [23]. For instance, the tobacco industry used donations to cultural organizations to help enlist their support against a proposed public smoking ban in New York City [86]. From that perspective, PepsiCo's Refresh Project represents $20 million in donations to community groups who publicly praise the company [73], and may be recruited to help oppose future regulatory initiatives. Moreover, PepsiCo and Coca-Cola are members of the American Beverage Association (ABA), an industry trade group that has aggressively lobbied against taxes on SSBs [87]. Following a trademark tobacco industry tactic, the soda companies and the ABA are members of the front group “Americans Against Food Taxes,” which, despite its name, is primarily composed of food and beverage companies. The group has aired a $10 million TV campaign against taxing beverages and promoting individual responsibility as the remedy for obesity [88].

## Unlike Tobacco, Soda CSR Explicitly Seeks Sales, and Sales to Youth

In contrast to the actions of Big Tobacco, soda industry CSR initiatives are explicitly and aggressively profit-seeking. Soda companies use CSR to tout their concern for the health and well-being of youth while simultaneously cultivating brand loyalty. The stated goal of PepsiCo's flagship Refresh Project is to increase long-term sales [73],[89] by engaging youth in the initiatives [69] and to build loyalty by associating PepsiCo with benevolent, worthwhile ventures. According to PepsiCo, after just nine months, the Refresh Project is an overwhelming success: “With over 2.8 billion (with a “B”!) earned media impressions, the project exceeded our internal benchmarks early in the year and we've seen an improvement in key brand health metrics. Crucial to PepsiCo's bottom line, when Millennials, the campaign's key demographic target, know about the Project their purchase intent goes up” [90]. Such soda CSR programs focus strategically on this cohort of 11- to 31-year-olds [71] to build brand preferences from an early age and create a climate in which drinking soda is viewed as a natural, frequent activity.

Soda companies also benefit from sponsorship of youth-oriented community organizations in ways that are unavailable to tobacco companies, which must avoid appearing to attract young people as a condition of the MSA [91]. Soda companies' marketing to youth is not similarly constrained, and soda CSR campaigns target youth in schools or community centers. The use of cause marketing and new media facilitates the companies' connection to youth. For instance, Coke uses its Spark Your Park program, with heavy emphasis on Facebook and Twitter engagements online, to promote its Sprite product while donating funds to neglected recreation facilities [92]. Moreover, these CSR campaigns provide a mechanism for soda companies to circumvent pledges not to market in schools [93],[94],[95]. While soda companies agreed to remove full-calorie drinks from U.S. schools, CSR programs like the Refresh Project keep the brand in front of young people with promises of grants for children's schools, parks, or other programs.

## Soda Is Employing CSR Sooner Than Big Tobacco

The overall goal for the tobacco industry's CSR strategy has been to normalize its products and its corporate image [96],[97],[98], but it has struggled as public health advocates have denormalized tobacco use and challenged tobacco companies trying to rehabilitate their images. Historically, advocates countered such campaigns by stigmatizing smoking [99]. Now, denormalization characterizes the corporation's activities as a disease vector [100], and highlights the disingenuous use of CSR [101]. Such industry denormalization refutes the tobacco companies' argument that they are like any other legitimate industry [20], builds support for stronger regulation, and helps deter and reduce adolescent [102], young adult [103], and adult smoking [104],[105].

The soda industry appears to be improving upon Big Tobacco's CSR strategy by acting sooner [28]. Although the tobacco industry responded to critics in 1954 with the nationwide newspaper advertisement “A Frank Statement to Cigarette Smokers” [106], decades lapsed between the public's outcry regarding tobacco and when the industry mounted concerted CSR campaigns [107]. While soda companies may not face the level of social stigmatization or regulatory pressure that now confronts Big Tobacco, concern over soda and the obesity epidemic is growing. The World Health Organization [108] and the U.S. Surgeon General cited soda as a key contributor to obesity [109], U.S. First Lady Michelle Obama's Let's Move initiative prompted new company policies by soda marketers [110], and interest in soda taxes is growing [111],[112]. The soda companies are feeling this pressure. In 2009, Coca-Cola told its shareholders that “Increasing concern among consumers, public health professionals and government agencies of the potential health problems associated with obesity and inactive lifestyles represents a significant challenge to our industry” [113]. Unlike tobacco, at the first signs of soda denormalization soda companies quickly launched comprehensive, well-funded, international CSR campaigns that take advantage of social media.

## Implications for Public Health Advocates

Tobacco companies launched CSR campaigns to rehabilitate themselves with the public when their image had been tarnished [20]. Because the most comprehensive initiatives were introduced well after intense public outcry, however, their CSR efforts struggled to achieve their aims [42]. As soda denormalization is nascent, soda companies may enjoy benefits from CSR that Big Tobacco labored to accomplish. In addition to effectively preempting regulation and maintaining its favorable position with the public, the soda industry's CSR tactics may also entice today's young people to become brand-loyal lifetime consumers, an outcome that current social norms dictate Big Tobacco cannot explicitly seek.

Without sustained denormalization of soda, it will be harder for public health advocates to see why partnering with industry may further the companies' goals more than their own. While tobacco denormalization was facilitated by litigation, which used the discovery process to procure internal documents revealing the industry's duplicitous intent, it is possible to respond to the soda industry without a “smoking gun.” For example, one instance of tobacco industry denormalization that did not rely on internal documents was the revelation that PM spent more on publicizing its charitable efforts than it spent on the charities itself, which exposed the cynical nature of Big Tobacco's CSR [114]. The Refresh Project's $20 million price tag, and the statements from company representatives, give public health advocates a similar opportunity to argue that this is marketing, not philanthropy [115]. Such criticism appeared in a *Lancet* editorial, which stated that the U.K.'s Change4Life should have avoided “ill-judged partnerships with companies that fuel obesity” [116]. Research on the health harms of sugary beverages can help advocates name these products as one of the “biggest culprits” [117] behind the obesity crisis. Emerging science on the addictiveness [118] and toxicity [119] of sugar, especially when combined with the known addictive properties of caffeine found in many sugary beverages, should further heighten awareness of the product's public health threat similar to the understanding about the addictiveness of tobacco products.

Public health advocates must continue to monitor the CSR activities of soda companies, and remind the public and policymakers that, similar to Big Tobacco, soda industry CSR aims to position the companies, and their products, as socially acceptable rather than contributing to a social ill.

## Supporting Information

Alternative Language Abstract S1 Spanish translation of the abstract.
(DOCX)

## Abbreviations

CSR: corporate social responsibility
MSA: Master Settlement Agreement
PM: Philip Morris
SSB: sugar-sweetened beverage
