# Supplementary material for: Soda and Tobacco Industry Corporate Social Responsibility Campaigns: How Do They Compare?
Source: PLoS Med. 2012 Jun 19;9(6):e1001241. doi: 10.1371/journal.pmed.1001241 (PMC3378589; doi:10.1371/journal.pmed.1001241)
Supplement: Alternative Language Abstract S1 — Spanish translation of the abstract. (DOCX) [file pmed.1001241.s001.docx]

Spanish translation of abstract:

En la trayectoria de la implicación que bebidas azucarados son un factor en la crisis global de la obesidad, grandes fábricas de refrescos han diseñado campañas multinacionales costosas y detalladas para demostrar la responsabilidad social empresarial (RSE o *CSR* por su sigla en ingles).

Estas campañas se parecen a las de la industria de tabaco, la cual usaba RSE como un medio para concentrar la responsabilidad en los consumidores en vez de la empresa, aumentar la popularidad de la compañía y de sus productos, y prevenir regulación.

Respondiendo a preocupaciones de salud acerca de sus productos, compañías de refrescos parecen haber lanzado iniciativas de RSE antes de cuando lo hicieron las compañías de tabaco.

A diferencia de las campañas de RSE de tabaco, las campañas de RSE de empresas de refrescos tienen el propósito explícito de aumentar ventas, incluyendo ventas entre jóvenes.

Así como hicieron con tabaco, los defensores de salud pública tienen que exponer estas campañas por lo que de verdad son, y responder a las campañas de RSE con sus propias campañas para educar al público y a los políticos que toman decisiones acerca de los efectos de campañas de RSE y la enfermedad social causada por bebidas azucaradas.
